# Carriers of Care-Cultural Translation, Recognition, and Belonging Among International Medical Graduates in the NHS

**DOI:** 10.1192/bjo.2026.11076

**Published:** 2026-06-30

**Authors:** Anindya Kar

**Affiliations:** Cornwall Partnership NHS Foundation Trust, Bodmin, United Kingdom. Goldsmiths, University of London, London, United Kingdom

## Abstract

**Aims:**

International Medical Graduates (IMGs) are essential to the delivery of care in the UK National Health Service (NHS) but their work is often examined through workforce metrics rather than lived clinical practice. So far, this study presents one of the first in-depth UK-based ethnographic analyses of IMG doctors, examining how they negotiate professional belonging, authority, and recognition, and how cultural translation and plural clinical reasoning operate under conditions of rota pressure, visa constraint, and racialised evaluation.

**Methods:**

An interpretative qualitative study was conducted using ethnographic methods. Data were generated through 13 life-history interviews with IMG doctors working across NHS hospital settings, supported by reflexive field diaries and digital ethnography within IMG professional WhatsApp groups. Participants represented a range of specialties, grades, contract types, and intersectional identities. Data were analysed thematically using concepts from medical anthropology and sociology, with attention to recognition, translation practices, and institutional governance. Ethical approval was obtained from the relevant institutionalethics committee, all participants provided informed consent, and data were anonymised in accordance with governance and data protection requirements.

**Results:**

IMGs contributed more than clinical labour. Participants described sustained cultural translation work, rendering care credible across accents, professional rubrics, and patient moral worlds. Many enacted plural clinical rationalities, making small, evidence-tested adaptations to standard protocols that stabilised pressured services and supported patient trust. These practices were frequently unrecognised or misread as inefficiency. Professional recognition was unevenly distributed and patterned by race, accent, grade, and visa status, with service-heavy posts limiting access to protected training and clinical voice. Performance, strategic silence, and low-visibility resistance emerged as everyday tactics through which IMGs preserved dignity, mitigated risk, and advocated for patients.

**Conclusion:**

The NHS already functions as a global medical system but its infrastructures of recognition remain misaligned with this reality. Recognising cultural translation as clinical work and embedding safe mechanisms to evaluate plural practices could improve equity, patient safety, and workforce retention. Practical implications include protecting training in service-heavy roles, decoupling communication assessment from accent policing, embedding IMG-led forums into quality improvement processes, and designing visa-aware governance.

